# Supplementary material for: Using colony size to measure fitness in Saccharomyces cerevisiae
Source: PLoS One. 2022 Oct 13;17(10):e0271709. doi: 10.1371/journal.pone.0271709 (PMC9560512; doi:10.1371/journal.pone.0271709)
Supplement: S1 Table — Evolved strains are indicated by the name of their stress (COPR_A for copper and SaltA for sodium), and the percent lethal limit in which they were raised (e.g. EH0_80 indicates an evolutionary history of environment fluctuation between 0 and 80 percent lethal stress). (PDF) [file pone.0271709.s001.pdf]

S1 Table. Strains used in this study.

Strain	Description	Source
COPR_A EH0; YJF4668	Evolved from ancestors d1E1 and d2E1 in CM.	[34]
COPR_A EH0_80; YJF4669	Evolved from ancestors d1E9 and d2E9 in CM and CM + 6.4 µM $CuSO_4$ on alternating days	
COPR_A EH80; YJF4670	Evolved from ancestors d1H5 and d2H5 in CM + 6.4 µM $CuSO_4$	
SaltA EH0; YJF4671	Evolved from ancestors d1E1 and d2E1 in CM	
SaltA EH0_80; YJF4672	Evolved from ancestors d1E9 and d2E9 in CM and CM + 274 mM NaCl on alternating days	
SaltA EH80; YJF4673	Evolved from ancestors d1H5 and d2H5 in CM + 274 mM NaCl	
Ancestor d1E1; YJF4674	Derived from mating YJF153 (MATa, HO::dsdAMX4 with barcoded kanMX deletion casettes from the MoBY plasmid collection) and YJF154 (MATalpha, HO::dsdAMX4). Both parents are derivatives of an oak tree strain, YPS163.	
Ancestor d2E1; YJF4675		
Ancestor d1E9; YJF4676		
Ancestor d2E9; YJF4677		
Ancestor d1H5; YJF4678		
Ancestor d2H5; YJF4679		
YJF4604	YJF1389 (MATa, HO::YFP-NAT, ura3-140) mated to YJF154 (MATalpha, HO:dsdAMX4). Both parents are derivatives of YPS163.	This study, [35]

Evolved strains are indicated by the name of their stress (COPR_A for copper and SaltA for sodium), and the percent lethal limit in which they were raised (e.g. EH0_80 indicates an evolutionary history of environment fluctuation between 0 and 80 percent lethal stress).
