# Supplementary material for: Using colony size to measure fitness in Saccharomyces cerevisiae
Source: PLoS One. 2022 Oct 13;17(10):e0271709. doi: 10.1371/journal.pone.0271709 (PMC9560512; doi:10.1371/journal.pone.0271709)
Supplement: S2 Table — The root mean square error (RMSE) and variance in fitness explained by strain (R2) are derived from the fitness difference ~ evolved strain relationship at each stress concentration using the data shown in Figs 2 and 3. Minimum detectable fitness differences is from power analysis at 0.80 power and 0.95 significance. (PDF) [file pone.0271709.s002.pdf]

S2 Table. Summary of measurement properties from the competitive fitness and colony size assays.

Assay	Stress	Stress Concentration	Sample Number	RMSE	R^2	Smallest Detected Fitness Difference	Minimum Detectable Fitness Difference
Colony Size	$CuSO_4$	0 μM	32	0.0085	0.629	0.0026	0.0043
		10 μM		0.0086	0.485	0.0030	0.0044
		20 μM		0.0281	0.909	0.0130	0.0144
		30 μM		0.1128	0.859	0.0451	0.0577
		40 μM		0.2138	0.869	1.1021	0.1093
	NaCl	0 mM		0.0107	0.518	0.0041	0.0055
		10 mM		0.0108	0.430	0.0024	0.0055
		50 mM		0.0076	0.678	0.0025	0.0039
		100 mM		0.0087	0.847	0.0037	0.0044
		150 mM		0.0153	0.685	0.0079	0.0078
		200 mM		0.0121	0.743	0.0050	0.0062
		300 mM		0.0176	0.724	0.0361	0.0090
		400 mM		0.0294	0.718	0.0147	0.0150
		500 mM		0.0171	0.836	0.0119	0.0088
		600 mM		0.0190	0.874	0.0182	0.0097
		800 mM		0.0404	0.565	0.0145	0.0207
		1000 mM		0.0239	0.498	0.0131	0.0122
		1200 mM		0.0461	0.244	0.0311	0.0236
Comp-etitive Fitness	CM	0 μM	7	0.0107	0.903	0.0221	0.0136
	$CuSO_4$	5 μM		0.0168	0.779	0.0050	0.0214
	NaCl	103 mM		0.0145	0.724	0.0148	0.0185

The root mean square error (RMSE) and variance in fitness explained by strain (R^2) are derived from the fitness difference ~ evolved strain relationship at each stress concentration using the data shown in Figs 2 and 3. Minimum detectable fitness differences is from power analysis at 0.80 power and 0.95 significance.
