# Supplementary material for: Using colony size to measure fitness in Saccharomyces cerevisiae
Source: PLoS One. 2022 Oct 13;17(10):e0271709. doi: 10.1371/journal.pone.0271709 (PMC9560512; doi:10.1371/journal.pone.0271709)
Supplement: S3 Table — (PDF) [file pone.0271709.s003.pdf]

S3 Table. Reagents and equipment used in the experiments.

Reagent	Description
Ammonium Sulfate	CAS # 7783-20-2, Fisher Chemical A702
Bacto Peptone	BD 211820
Bacto Yeast Extract	BD 212720
Cupric Sulfate	CAS # 7758-98-7, Fisher BioReagents BP346
Dextrose	CAS # 50-99-7, Fisher Chemical D16
Difco Agar, Bacteriological	BD 214510
Drop-out Mix Complete	Without yeast nitrogen base, US Biological D9515
Sodium Chloride	CAS # 7467-14-5, Fisher Chemical S271
Yeast Nitrogen Base	Without amino acids and ammonium sulfate, BD 233520
**Equipment and Software**	
BD Biosciences Accuri C6	
BioTek Instruments Epoch 2	
Singer Instruments Rotor	
Singer Instruments PhenoBooth	
Singer Instruments PhenoSuite v. 2.20.504.1	
