# Supplementary material for: Using colony size to measure fitness in Saccharomyces cerevisiae
Source: PLoS One. 2022 Oct 13;17(10):e0271709. doi: 10.1371/journal.pone.0271709 (PMC9560512; doi:10.1371/journal.pone.0271709)
Supplement: S1 Fig — Colony photographs and size distributions by layer for days 1–3 associated with Fig 1B–1E. Evolved and ancestor strains were arrayed in a randomized pattern on CM agar plates either left intact or trimmed immediately after printing (A and B, respectively). Boxes indicate the 95% confidence interval bisected by the mean. Stars indicate layers that are significantly different from the center (FDR < 0.05, t-test). (PDF) [file pone.0271709.s004.pdf]

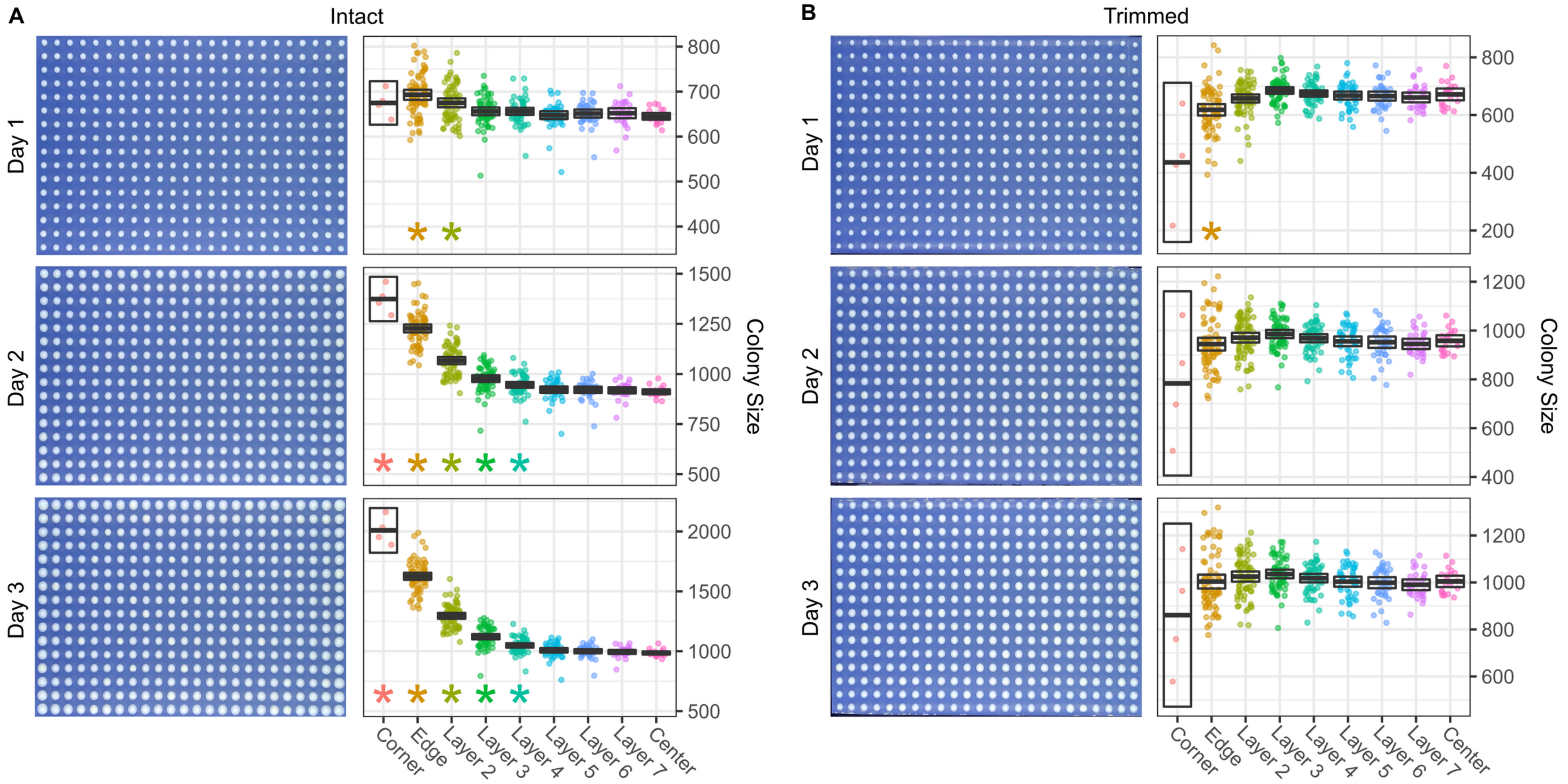

S1 Figure. Edge effects over time. Colony photographs and size distributions by layer for days 1-3 associated with Fig 1B-E. Evolved and ancestor strains were arrayed in a randomized pattern on CM agar plates either left intact or trimmed immediately after printing (A and B, respectively). Boxes indicate the 95% confidence interval bisected by the mean. Stars indicate layers that are significantly different from the center (FDR < 0.05, t-test).
