# Supplementary material for: Using colony size to measure fitness in Saccharomyces cerevisiae
Source: PLoS One. 2022 Oct 13;17(10):e0271709. doi: 10.1371/journal.pone.0271709 (PMC9560512; doi:10.1371/journal.pone.0271709)
Supplement: S2 Fig — Examples of colony size, estimated number of cells, generations and fitness differences over four days with corresponding photographs (A). Colony size areas (pixels) were recorded for four consecutive days after pinning and the size on day 0 was set to 57 (B). Colony size was converted to cell number using the experimentally derived log-log relationship (Fig 1A) and plotted as a function of ancestor generations (C). Fitness differences were determined from the difference in regression slopes (from panel C) between each evolved strain and the average of its ancestor pair as a function of elapsed days (D). (PDF) [file pone.0271709.s005.pdf]

A

	Ancestor d1H5	Ancestor d2H5	COPR_A EH80	SaltA EH80
Day 1	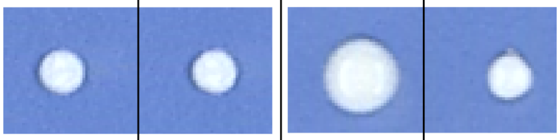			
Size	237	231	687	247
Cells	4.667E+06	4.495E+06	2.217E+07	4.958E+06
Average Ancestor Generations			2.9826	
log(Cells)~Generations	0.699	0.687	1.222	0.720
Fitness Difference	0.0063	-0.0063	0.5287	0.0266
Day 2	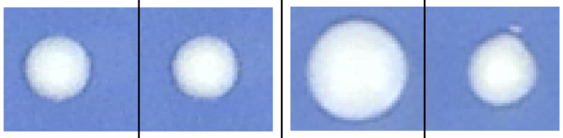			
Size	500	496	1292	595
Cells	1.392E+07	1.376E+07	5.589E+07	1.796E+07
Average Ancestor Generations			4.578	
log(Cells)~Generations	0.695	0.691	1.027	0.746
Fitness Difference	0.0019	-0.0019	0.3334	0.0530
Day 3	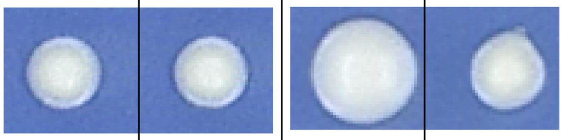			
Size	692	683	1524	780
Cells	2.240E+07	2.198E+07	7.117E+07	2.670E+07
Average Ancestor Generations			5.259	
log(Cells)~Generations	0.695	0.692	0.934	0.737
Fitness Difference	0.0014	-0.0014	0.2411	0.0438
Day 4	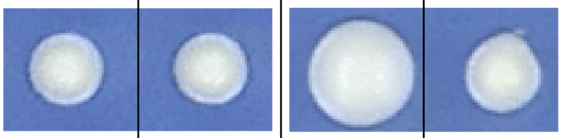			
Size	714	705	1567	809
Cells	2.346E+07	2.302E+07	7.413E+07	2.816E+07
Average Ancestor Generations			5.3255	
log(Cells)~Generations	0.694	0.692	0.902	0.734
Fitness Difference	0.0012	-0.0012	0.2084	0.0411

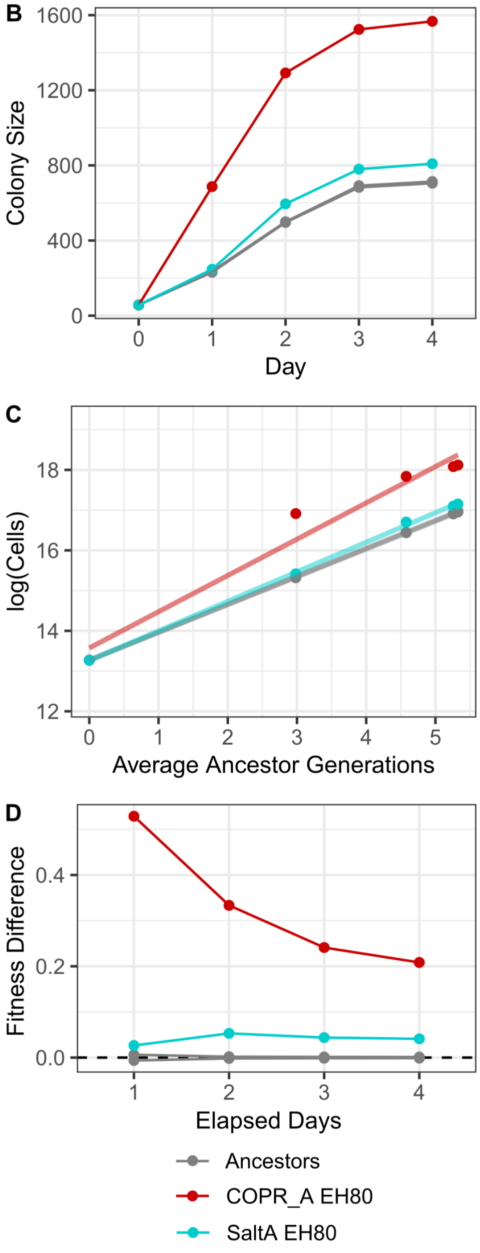

S2 Figure. Estimating fitness differences from colony size. Examples of colony size, estimated number of cells, generations and fitness differences over four days with corresponding photographs (A). Colony size areas (pixels) were recorded for four consecutive days after pinning and the size on day 0 was set to 57 (B). Colony size was converted to cell number using the experimentally derived log-log relationship (Fig 1A) and plotted as a function of ancestor generations (C). Fitness differences were determined from the difference in regression slopes (from panel C) between each evolved strain and the average of its ancestor pair as a function of elapsed days (D).
