# Supplementary material for: Using colony size to measure fitness in Saccharomyces cerevisiae
Source: PLoS One. 2022 Oct 13;17(10):e0271709. doi: 10.1371/journal.pone.0271709 (PMC9560512; doi:10.1371/journal.pone.0271709)
Supplement: S3 Fig — Pure cultures were used to determine the single stress concentrations for competitive growth assays. The area under the curve, carrying capacity, time lag and maximum growth rate for different concentrations of copper and sodium were determined from growth curves of each strain (n = 1). Growth measures are shown relative to growth in CM without added copper or sodium. Dotted lines indicate the concentration chosen for competitive fitness assays. (PDF) [file pone.0271709.s006.pdf]

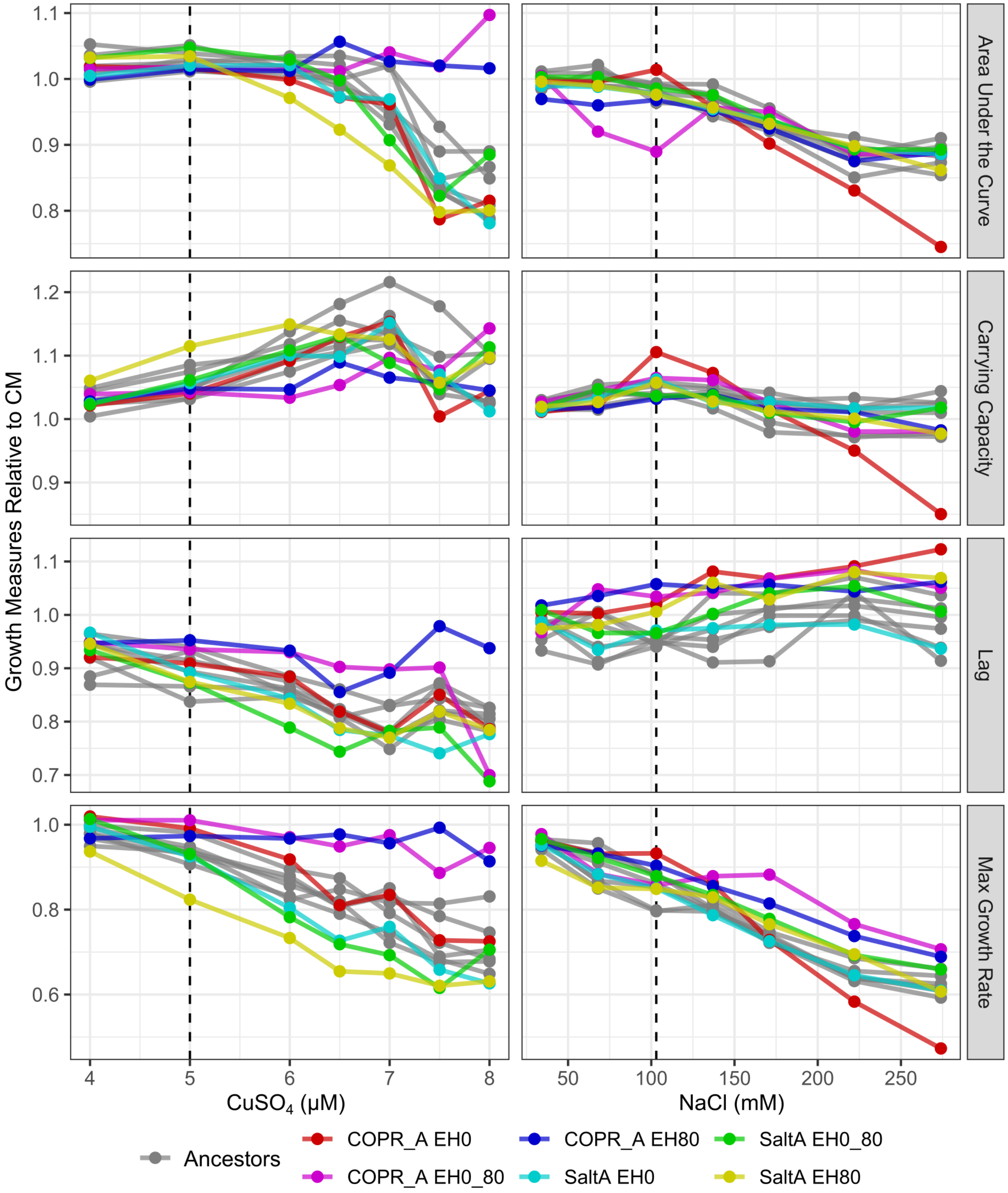

S3 Figure. Liquid growth curve characteristics of evolved and ancestor strains. Pure cultures were used to determine the single stress concentrations for competitive growth assays. The area under the curve, carrying capacity, time lag and maximum growth rate for different concentrations of copper and sodium were determined from growth curves of each strain (n = 1). Growth measures are shown relative to growth in CM without added copper or sodium. Dotted lines indicate the concentration chosen for competitive fitness assays.
