# Supplementary material for: Using colony size to measure fitness in Saccharomyces cerevisiae
Source: PLoS One. 2022 Oct 13;17(10):e0271709. doi: 10.1371/journal.pone.0271709 (PMC9560512; doi:10.1371/journal.pone.0271709)
Supplement: S4 Fig — Gating parameters were determined from the pooled events of all runs pertaining to a single day and stress. The first gate excluded events outside the 99% ellipse from log10 SSC-A (side scatter area) vs. log10 FSC-A (forward scatter area)(A). This was followed by event exclusion from a 95% ellipse on a FSC-H (forward scatter height) vs. width (B). The YFP gate was manually set at the midpoint between the two largest populations on a log10 YFP-A histogram and kept at a constant value throughout all experiments (C). Example YFP-A histograms after gating showing the competition between an ancestor strain and the YFP-expressing reference strain, and a reference-only control consisting of YJF4606 alone (D). Note the small proportion of the events exhibited fluorescent signalling below the YFP gate in these reference only controls (1–19%) which are accounted for as the false negative fraction (Eq 1). (PDF) [file pone.0271709.s007.pdf]

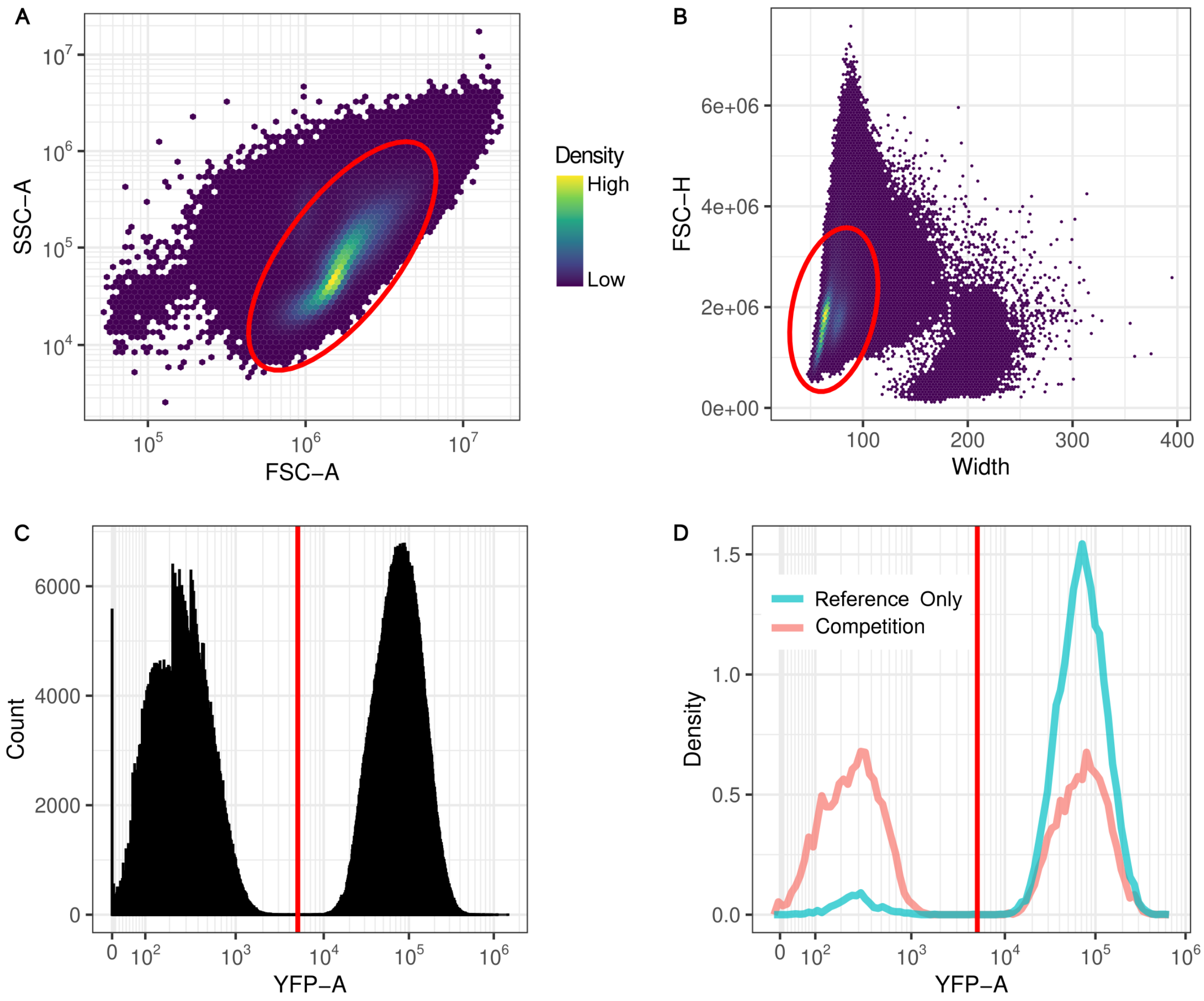

S4 Figure. Gating scheme used to measure the proportion of YFP cells for the competitive fitness assays. Gating parameters were determined from the pooled events of all runs pertaining to a single day and stress. The first gate excluded events outside the 99% ellipse from log10 SSC-A (side scatter area) vs. log10 FSC-A (forward scatter area)(A). This was followed by event exclusion from a 95% ellipse on a FSC-H (forward scatter height) vs. width (B). The YFP gate was manually set at the midpoint between the two largest populations on a log10 YFP-A histogram and kept at a constant value throughout all experiments (C). Example YFP-A histograms after gating showing the competition between an ancestor strain and the YFP-expressing reference strain, and a reference-only control consisting of YJF4606 alone (D). Note the small proportion of the events exhibited fluorescent signalling below the YFP gate in these reference only controls (1-19%) which are accounted for as the false negative fraction (Equation 1).
