# Supplementary material for: Using colony size to measure fitness in Saccharomyces cerevisiae
Source: PLoS One. 2022 Oct 13;17(10):e0271709. doi: 10.1371/journal.pone.0271709 (PMC9560512; doi:10.1371/journal.pone.0271709)
Supplement: S5 Fig — Power and number of replicates needed for competitive fitness (dashed lines, labeled “C.F.”) and colony size assays (solid lines) for CM only (A and B) and various concentrations of copper (C and D) and sodium (E and F). Power as a function of fitness difference was derived from each assay’s measured performance (A, C and E). The number of replicates needed to achieve 0.8 power at 0.95 significance is plotted as a function of fitness difference (B, D and F). (PDF) [file pone.0271709.s008.pdf]

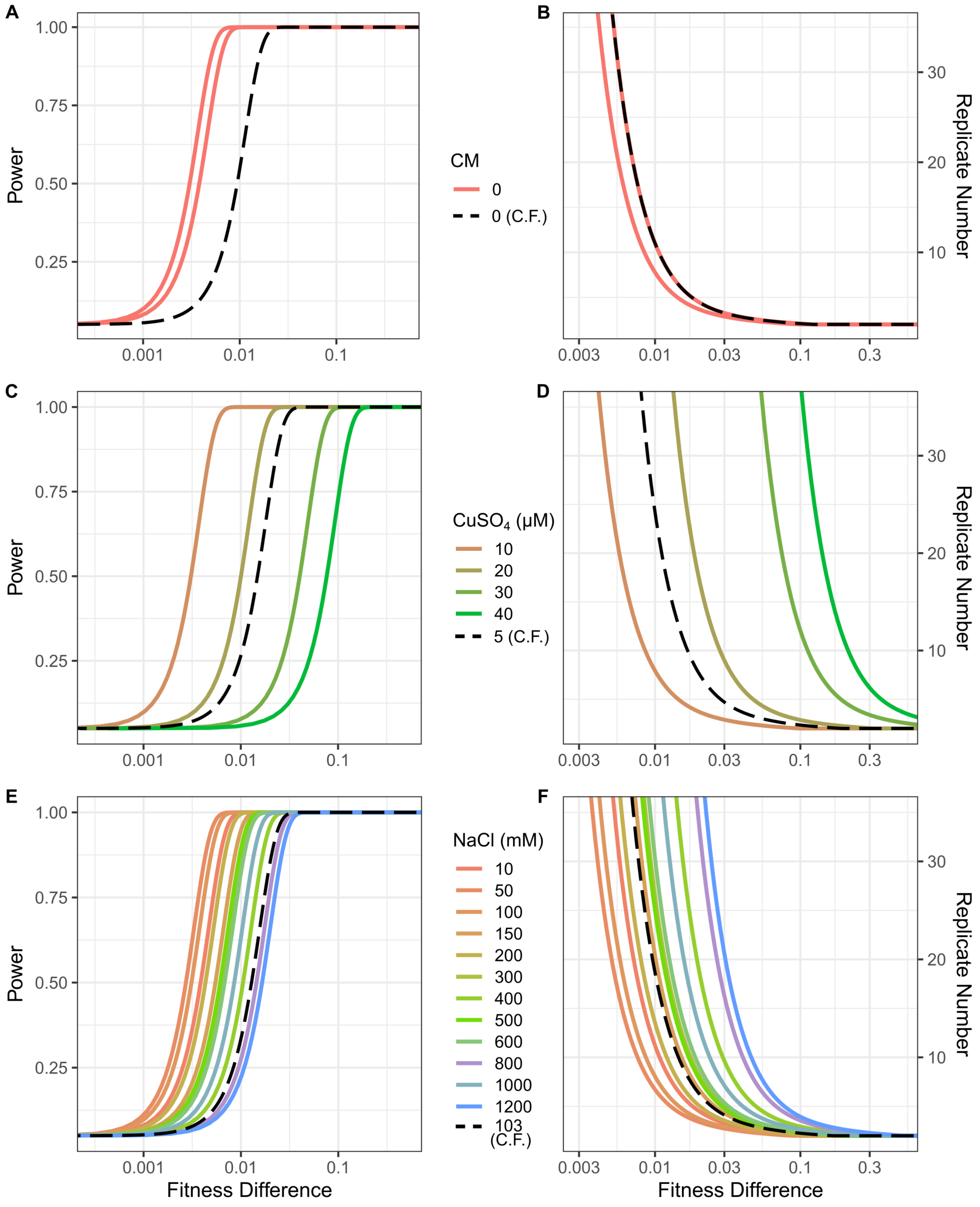


**S5 Figure. Power analyses of competitive fitness and colony size assays.** Power and number of replicates needed for competitive fitness (dashed lines, labeled “C.F.”) and colony size assays (solid lines) for CM only (A and B) and various concentrations of copper (C and D) and sodium (E and F). Power as a function of fitness difference was derived from each assay’s measured performance (A, C and E). The number of replicates needed to achieve 0.8 power at 0.95 significance is plotted as a function of fitness difference (B, D and F).
