# Supplementary material for: Using colony size to measure fitness in Saccharomyces cerevisiae
Source: PLoS One. 2022 Oct 13;17(10):e0271709. doi: 10.1371/journal.pone.0271709 (PMC9560512; doi:10.1371/journal.pone.0271709)
Supplement: S6 Fig — Variance explained (R2) is shown for intact and trimmed plates arrayed on CM plates in 96, 384 or 1536 density formats. All colonies are of ancestor strain YJF4679 and corner colonies were excluded. (PDF) [file pone.0271709.s009.pdf]

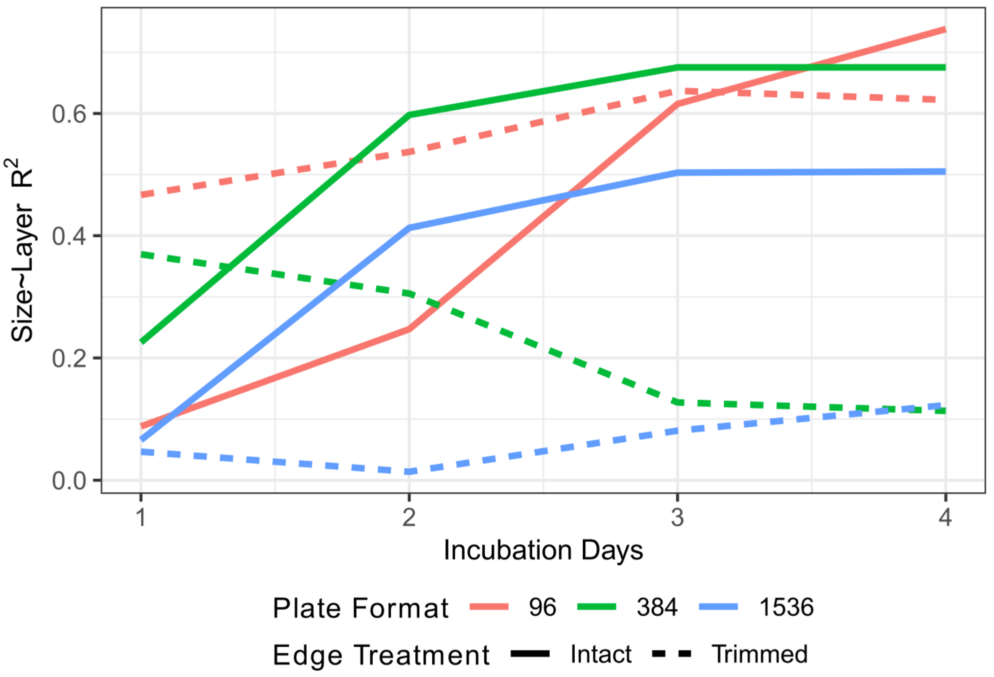

S6 Figure. The proportion of variance explained by layer as a function of incubation time. Variance explained (R^2) is shown for intact and trimmed plates arrayed on CM plates in 96, 384 or 1536 density formats. All colonies are of ancestor strain YJF4679 and corner colonies were excluded.
