# Supplementary material for: Using colony size to measure fitness in Saccharomyces cerevisiae
Source: PLoS One. 2022 Oct 13;17(10):e0271709. doi: 10.1371/journal.pone.0271709 (PMC9560512; doi:10.1371/journal.pone.0271709)
Supplement: S7 Fig — Colonies are after 4 days growth from trimmed and intact CM plates with no normalization and normalization by row and column mean, row and column median, or layer. Numbers in the upper right indicate the size variance explained by strain (colony size ~ strain). Boxes show the 95% confidence interval bisected by the mean with stars indicating layers that significantly differed from the center layer (FDR < 0.05, two sample t-test). (PDF) [file pone.0271709.s010.pdf]

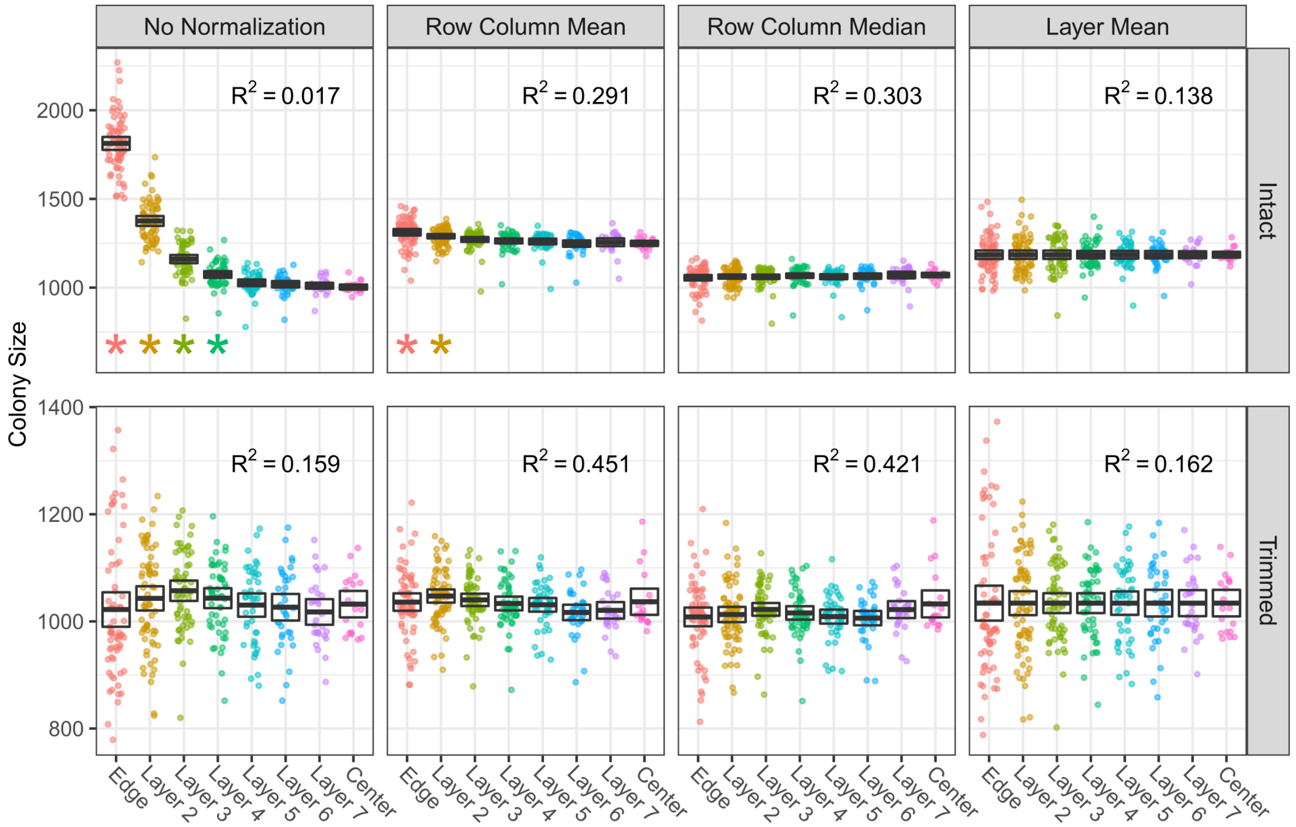

S7 Figure. Colony sizes from various normalization methods. Colonies are after 4 days growth from trimmed and intact CM plates with no normalization and normalization by row and column mean, row and column median, or layer. Numbers in the upper right indicate the size variance explained by strain (colony size ~ strain). Boxes show the 95% confidence interval bisected by the mean with stars indicating layers that significantly differed from the center layer (FDR < 0.05, two sample t-test).
