# Supplementary material for: Using colony size to measure fitness in Saccharomyces cerevisiae
Source: PLoS One. 2022 Oct 13;17(10):e0271709. doi: 10.1371/journal.pone.0271709 (PMC9560512; doi:10.1371/journal.pone.0271709)
Supplement: S8 Fig — Variance explained by strain (R2) before and after row and column mean normalization for either intact or trimmed plates containing various concentrations of sodium (left) or trimmed plates with copper (right). (PDF) [file pone.0271709.s011.pdf]

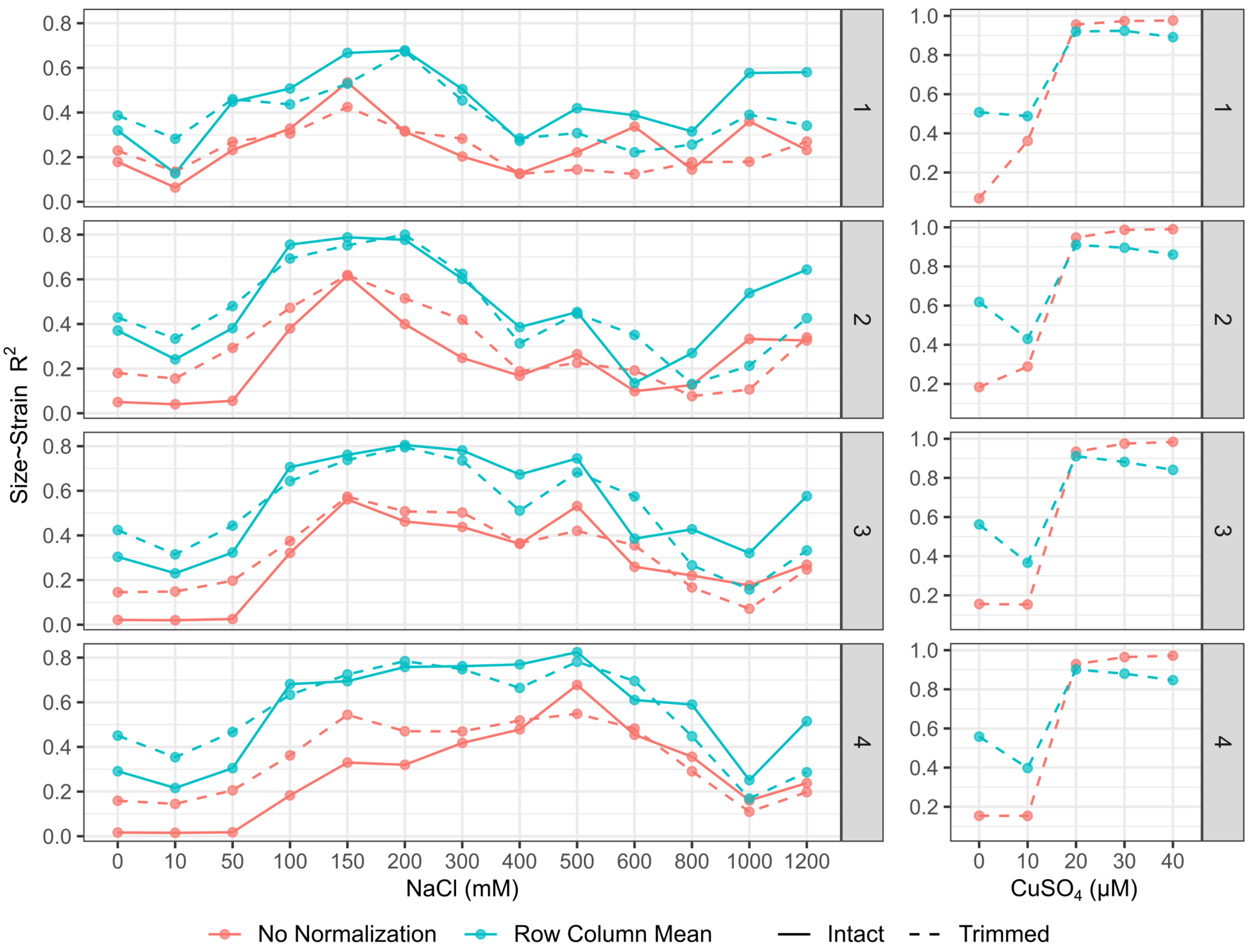

S8 Figure. Colony size variance explained by strain after normalization. Variance explained by strain (R^2) before and after row and column mean normalization for either intact or trimmed plates containing various concentrations of sodium (left) or trimmed plates with copper (right).
