# Supplementary material for: Using colony size to measure fitness in Saccharomyces cerevisiae
Source: PLoS One. 2022 Oct 13;17(10):e0271709. doi: 10.1371/journal.pone.0271709 (PMC9560512; doi:10.1371/journal.pone.0271709)
Supplement: S9 Fig — Points in black indicate that independent experiments resulted in overlapping 95% confidence intervals whereas points in red did not. Green boxes indicate the locations of the insets shown in each plot, the y = x line is shown in black. (PDF) [file pone.0271709.s012.pdf]

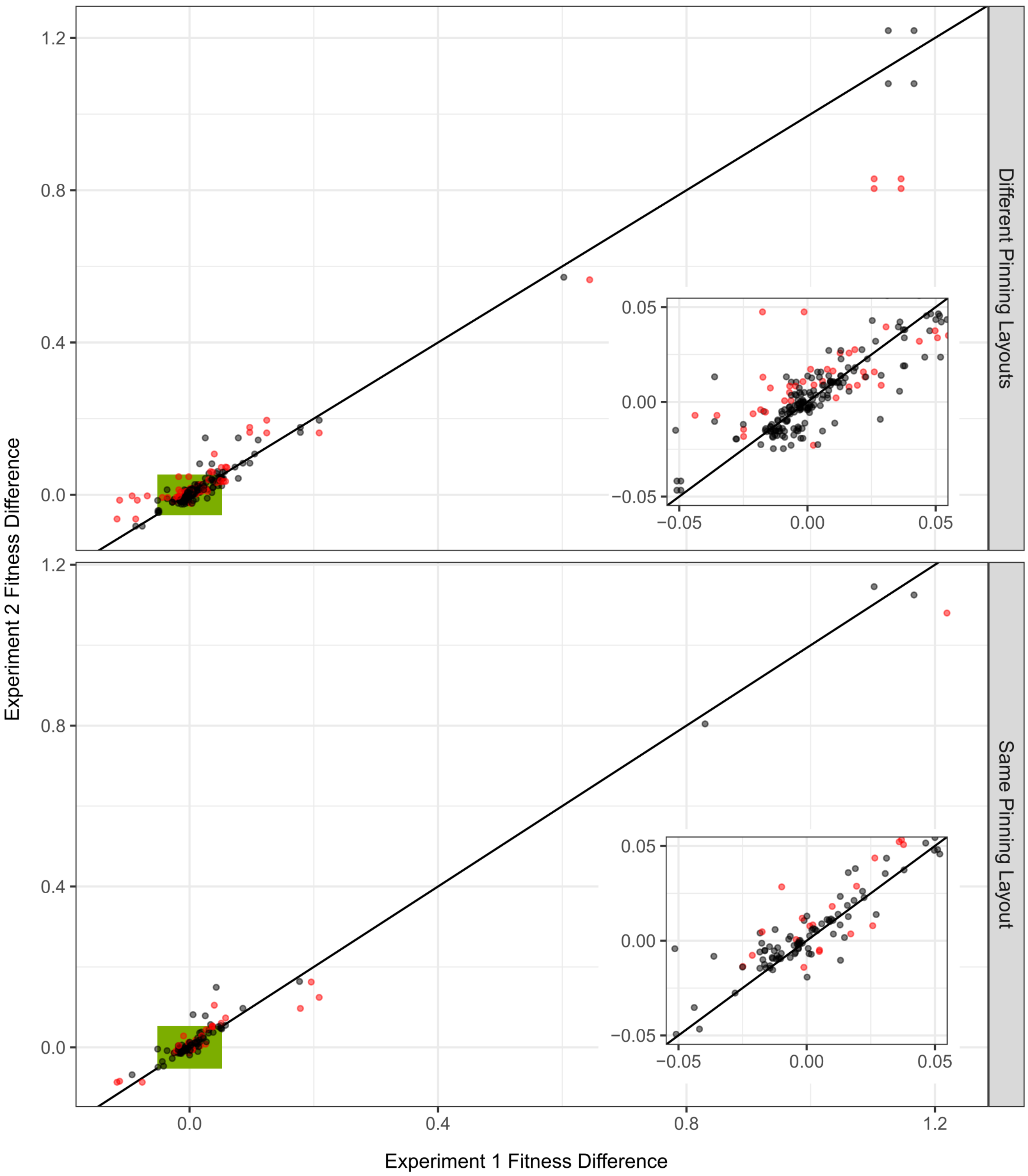

S9 Figure. Fitness differences estimated from independent experiments with either same or different pinning layouts. Points in black indicate that independent experiments resulted in overlapping 95% confidence intervals whereas points in red did not. Green boxes indicate the locations of the insets shown in each plot, the y=x line is shown in black.
