# Supplementary material for: Using colony size to measure fitness in Saccharomyces cerevisiae
Source: PLoS One. 2022 Oct 13;17(10):e0271709. doi: 10.1371/journal.pone.0271709 (PMC9560512; doi:10.1371/journal.pone.0271709)
Supplement: S10 Fig — The log of colony size ratios from Fig 3 were calculated by dividing the colony size of each evolved replicate by the average size of its respective ancestors on copper (A) and sodium (B) plates. The location of the inset is indicated by the black box. (PDF) [file pone.0271709.s013.pdf]

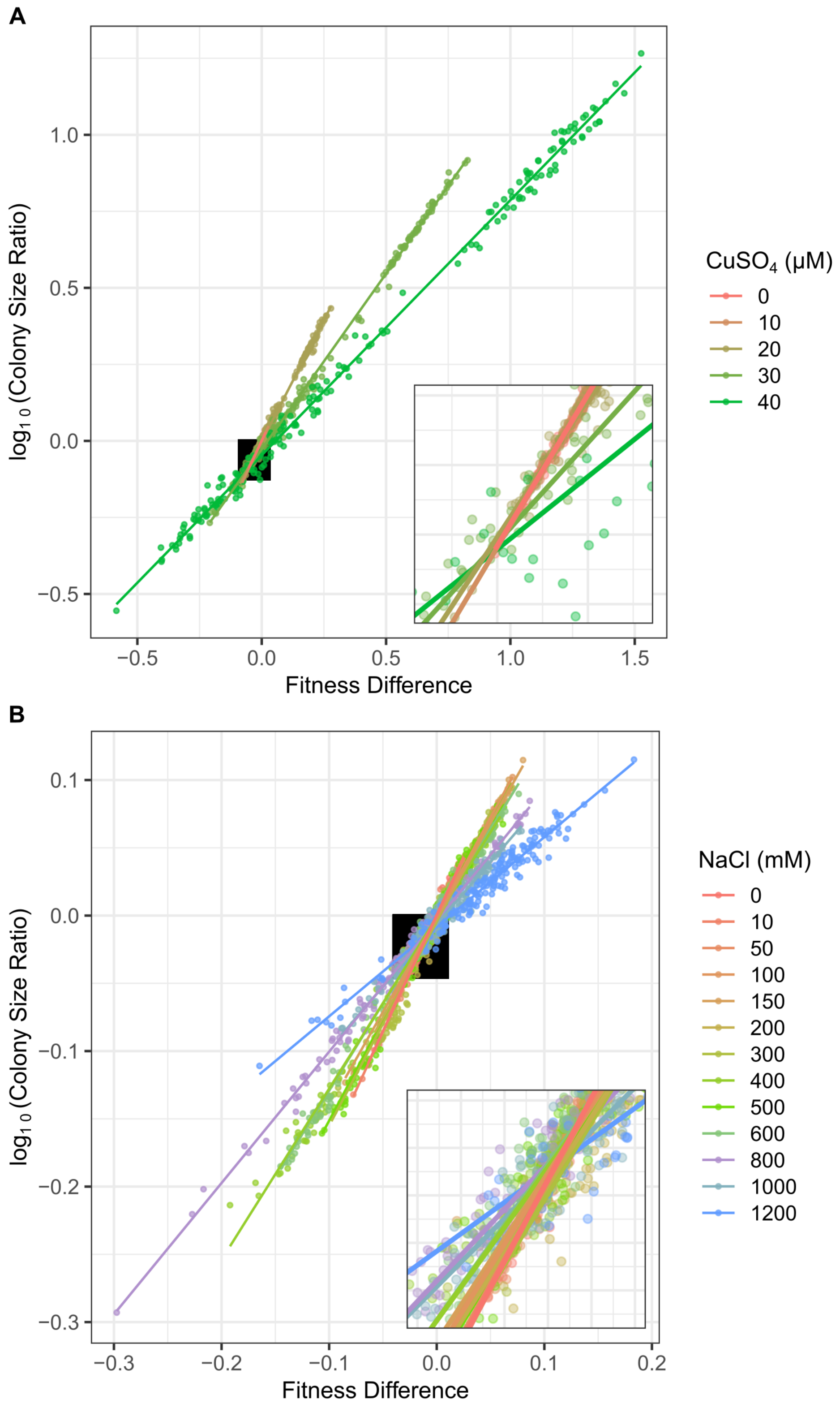

S10 Figure. Comparison of fitness differences and colony size ratios. The log of colony size ratios from Fig 3 were calculated by dividing the colony size of each evolved replicate by the average size of its respective ancestors on copper (A) and sodium (B) plates. The location of the inset is indicated by the black box.
