# Supplementary material for: Using colony size to measure fitness in Saccharomyces cerevisiae
Source: PLoS One. 2022 Oct 13;17(10):e0271709. doi: 10.1371/journal.pone.0271709 (PMC9560512; doi:10.1371/journal.pone.0271709)
Supplement: Attachment — Submitted filename: review_response.pdf [file pone.0271709.s015.pdf]

Reviewer response

Reviewer #1: In the present manuscript, Miller et al. have reported colony size as a metric to measure differences in relative fitness. There must be a broad audience for such a study given its high-throughput nature. However, there are some points which need to be addressed:

Major points

• Authors checked fitness through colony size using an image processing method. Do the authors only consider pixel count? since (presumably) images were taken in 2D space, pixel intensity could have improved the robustness of the methodology.

We did not examine pixel intensity, but we agree that based on prior studies this could improve the robustness of the method. Prior methods have also shown benefits of high density temporal imaging with optional phloxine B staining (Pyphe), and using a grid of control colonies to accurately eliminate spatial biases (Scan-o-matic & LI Detector). Our goal was not to develop an improved measurement of colony size, but rather to test whether colony size is sensitive enough to detect 1% fitness differences (per generation) that arose during experimental evolution. To accomplish this we opted for what we considered to be a straightforward normalized measure of colony size over four days (although once we observed the improvement of agar trimming, we kept this procedure). We have modified parts of the abstract/introduction/discussion to clarify our focus and we hope our work helps justify the use of other more sophisticated colony size methods to measure small fitness changes.

• Authors have run very different number of replicates for colony size and competitive fitness (32 vs 7). This is a huge difference. I also do not understand what n <= 32 (line 319) and ~32 (S2 Table) mean. It seems there were different number of replicates in colony size experiments. These differences can affect the power (which is one of the author's metric to evaluate and compare their results)

We generated power plots as a function of sample size to make colony size and competitive fitness comparable for a given number of replicates (Fig S5). The number of replicates on the plate was 32 for colony size, but was less than 32 due to missing data caused by eliminating corner colonies and a small number of colonies that were flagged during the QC process. This was not clear in the manuscript and we addressed this by using 32 to indicate the study design sample size.

• Was building the linear regression model (log10(Cells)=log10(Size)*1.

The linear regression is based on the standard form y = mx+b, where m = slope and b = intercept, y=log10(cells) and x=log10(size). We described this in the manuscript as "log10(Cells)=log10(Size)*1.46397+3.19251". We hope this is sufficiently clear.

• How would authors explain variability in RMSE and R2 values (S2 Table)? What is the biological reason. As you would see in following plot, there is no trend:
See the attached figure

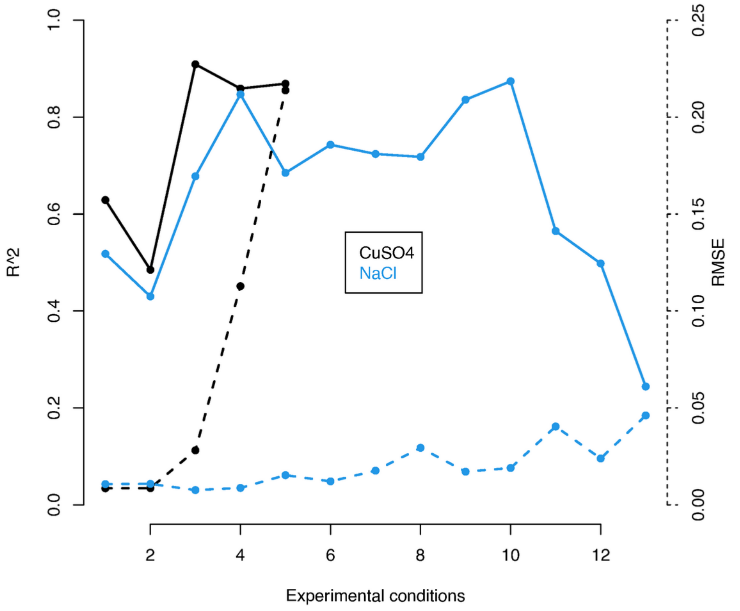

RMSE shows the error and R^2 shows the variance explained by strain. R^2 depends on how large the differences are between strains relative to how large differences are within strain replicates. Without stress (CM medium) R^2 is low because differences between strains are small relative to error, but R^2 increases with stress due to larger differences between strains. This can also be seen in Fig 3. RMSE shows that error is slightly increasing for salt, but increases rather dramatically for copper stress. One technical explanation is row/column normalization, which may increase error when most strains have no colonies (high copper stress in particular) and so each row or column only has a few values with which to normalize. There may be biological reasons why RMSE differs as well.

• As authors mentioned, number of replicates influence power of a statistical model (line 315). The observed power in this manuscript could also be due to their experimental design (large number of replicates; 32).
I also could not think of any reasons to justify power by levels of stress conditions (lines 322-322).

We showed the effect of sample size on power in Fig S5. For most of the manuscript we compared RMSE since it is independent of sample size. We examined power for different levels of stress because RMSE (which power depends on) is not constant across stress

concentrations and we wanted to be as transparent as possible in representing the power of colony size assay.

• Fig. 4. Authors linked fitness assays of solid and liquid cultures using Pearson's r while the variability of the results (from no correlation to relative correlation and negative correlation) makes it hard to believe there is any relations.

We agree and discussed explanations, but believe the readers will want to see these results.

In B and C sections, compared stress levels are different (NaCl: 200 vs 103 and CuSO4: 40 vs 5) and accordingly it is hard to make any conclusions.

We chose to show the strongest correlation so the readers could see there was not a good fit.

• In abstract, it is stated that "colony size is as sensitive as competitive fitness assays grown in liquid medium". There is no explanation in the main text and no evidence to support this claim.

Thank you for pointing this out. We agree. Our intention was to say that colony size can detect small differences, as small as the 1% differences commonly detected in competitive assays. We modified the abstract to more accurately state "colony size is a sensitve measure of fitness, capable of detecting 1% differences."

Minor points

• To increase reproducibility, I strongly recommend the authors to provide a supplementary table mentioning all available information about chemicals and equipment used in their study.

We have include this in Table S3.

• I was wondering if authors tried Poisson regression (instead of log10 transformation) given the data-type (cell count and pixel count).

Thanks for this suggestion. We tried a poisson regression and found a very similar visual fit as the log10 regression (see red line in the figure below). McFadden's R-squared for the Poisson was 0.83 and the R-squared for the log10 regression was 0.97. These aren't really comparable numbers but based on the similar visual fit we used the log10 regression.

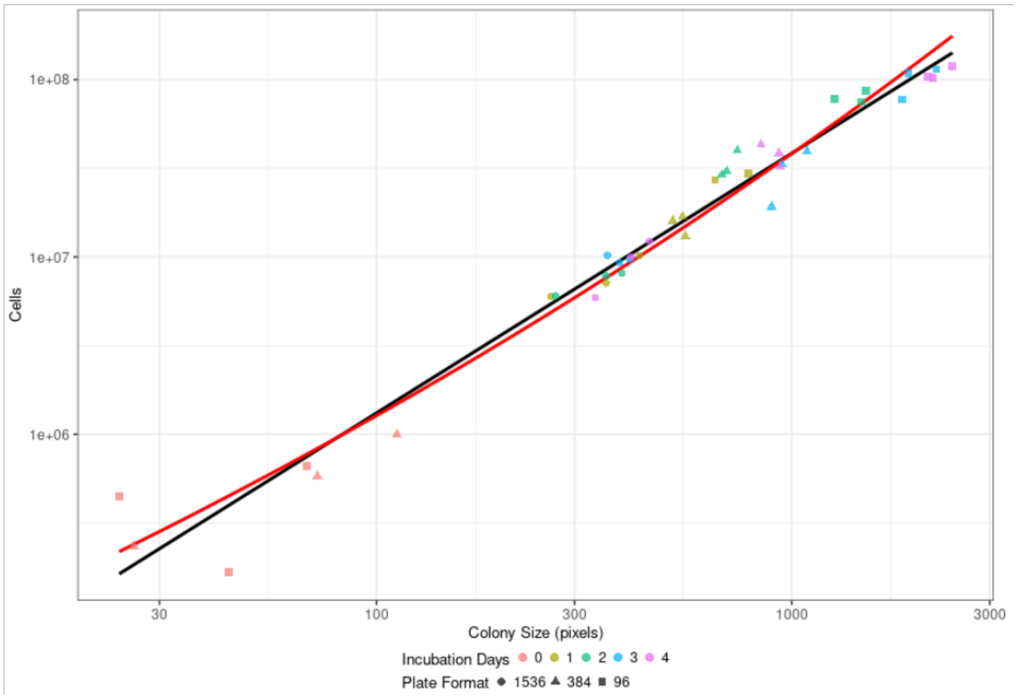

• Supplementary figures (such as Fig.1 and S1): the FDR threshold can be inferred to be 5% but it's better to be clearly mentioned (FDR = 5% instead of FDR-corrected). Is it one- or two-sided t-test?

We now use FDR < 0.05 throughout, which indicates the threshold and the possible range of values. We also now state the t-tests are two-sided in the methods.

• S1 Fig. A: The "intact" plates are inappropriately cropped. First column from right is cut out.

Corrected.

• "Various concentrations of $CuSO_4$ or NaCl" is mentioned several times in the text but there is no reference to S2 Table

We have added the concentrations for S3 Figure into the methods. The other places where this occurs (S5 and S8 Figure) have the concentrations in the legend. We added S2 Table to the first mention of varying concentrations in the methods. Note also that the academic editor asked for Table 2 to be included. The reference in the original submission was an error and should have been S2 Table. This has been corrected.

• Line 255: The sentence "Previously, the fitness of the evolved populations was measured in the presence and absence of sodium and copper stress." needs a reference.

Done.

• The word “Stress” in legend of Fig. 2 causes confusion: “CM means no stress”.

We have changed this to "Treatment".

• S8 Fig. Why there is no intact condition for cooper.

We only generated intact and trimmed plates for salt since these spanned the range of large to small colonies with large to small edge effects, respectively.

Reviewer #2: The authors report that by systematically obtaining the less significant fitness differences of "evolved strains" in solid medium and competitive culture, the measurement of growth (fitness) by colony size is sensitive enough to be comparable to competitive culture, which is generally considered a sensitive fitness test.

The authors have performed several interesting experiments. These include showing that removing the periphery of solid medium can reduce the peripheral effect, counting the number of cells in spotted colonies at various densities to determine the relationship between colony size and cell number, and the data obtained from these experiments are valuable.

On the other hand, the current format of the paper is limited to an introduction of the experimental methods performed by the authors. This is not enough to add novelty or scientific value to the paper. Most notably (as the authors mention in their paper), there have been numerous comparisons of fitness measures in colony size and other fitness tests (especially Baryshnikova 2010), and the differences between those methods and studies and the authors' method are not discussed. The difference between those methods and studies and the authors' method has not been discussed.

The distinction between our measurement of fitness and those of prior studies is subtle but important. We measured per generation fitness differences, which are needed to obtain selection coefficients. Selection coefficients determine the evolutionary dynamics of alleles under selection and are needed for comparison across studies and knowing the strength of selection (strong vs weak, Chevin 2011: doi:10.1098/rsbl.2010.0580). For example, given two populations of equal size but a 1% per generation fitness difference (i.e. selection coefficient of 1%), the ratio of the two population sizes (or colony size equivalents) is 0.990 after one generation but 0.905 after 10 generations. Previous studies estimate fitness from relative colony size or relative growth rate. This limits their comparison to per generation fitness differences estimated from competition assays. Even in the experimental evolution literature, the use of different measures of fitness makes it hard to compare fitness differences across studies, e.g. detecting a 1% vs a 2% difference (Chevin 2011: doi:10.1098/rsbl.2010.0580). We have added text to both the introduction and the discussion to make this point clear.

Baryshnikova et al. (2010) compared fitness based on colony size to three prior studies that measured fitness in liquid medium (Hughes et al. 2000, Deutschbauer et al. 2005, Breslow et al. 2008). They found correlations between 0.53 and 0.7. We have added this comparison as well as the correlation of 0.78 from Zackrisson et al. (2016) to our discussion of fitness based on colony size vs competitive growth.

In particular, as mentioned in Baryshnikova 2010, various corrections are made in the SGAtool. How does this differ from the authors' method? Is there any advantage to the authors' method? A full comparison and discussion of this would be necessary.

Baryshnikova et al. 2010 estimated fitness as a linear function of colony size with multiple scaling factors (Colony size = fitness * time * scaling parameters). Our measure of fitness is based on ln(cells) = fitness * generations, with cell number estimated from row and column normalized colony size. Thus, the two measures of fitness are not directly comparable (e.g. 1% fitness differences in one study could be equivalent to a 2% difference in the other study.

The colony size normalization procedures are comparable. We used a simple row and column normalization procedure. Baryshnikova et al. used scaling factors for plate (batch), row and column scaling with LOWESS smoothing, local spatial gradients from 7x7 colony grids, and growth of neighboring colonies. We now mention gridding and neighboring colonies in our discussion of spatial normalization. However, we don't feel it is appropriate to make a full comparison of our method to that of Baryshnikova et al or to the other studies of fitness based on colony size because i) we don't claim our fitness measurements are more accurate, and ii) there isn't a good metric for comparison. The best metric available to get some comparison across studies is measurement error (i.e. error in fitness), which is captured by RMSE and the coefficient of variation (CV) and was reported in a few of the studies. Although this isn't perfect, since it makes some assumptions about how the error changes with mean fitess, the RMSE and CV values are similar to one another ~2% with a range of 1-4%. We now also include the average CV (1.6%) from Baryshnikova et al. for single gene deletions. Thus, instead of pointing out differences between studies of colony size we suggest: "Thus, despite differences in experimental design most measures of fitness based on colony size appear well suited to detect small per generation fitness differences found in experimental evolution studies."

Minor comment:.

1) The results of individual liquid cultures, described in the methods, are not used in the main text. Why is there no mention of the difference in fitness between individual liquid cultures and colony size measurements?

We used the individual measurements to find appropriate concentrations for the competitive assays. However, the individual measurements had no replicates and we considered them to be too noisy for such a comparison. This noise is likely a consequence of our current plate reader (Epoch 2, BioTek) since we (using a different plate incubator/reader) and others have

obtained robust measures of liquid growth in the past.

2) The concentration of copper used in the "copper tolerance" test is only 1/1000th of that used in the previous report (PMID: 15059259). Where does this difference come from?

The prior study measured copper resistance on rich medium plates. In the current study we measured it on synthetic complete medium plates. Also, the ancestral oak strain is the most sensitive of all the strains in the previous study.
